# Design and Simulation of a Portable Five-Lead ECG Monitoring Device With Adaptive Filtering for Continuous Remote Cardiac Assessment

**DOI:** 10.7759/cureus.97201

**Published:** 2025-11-19

**Authors:** Drin Rrmoku

**Affiliations:** 1 Electrical Engineering, Independent Practice, Prishtina, ALB

**Keywords:** 5-lead ecg, adaptive filtering, cardiac assessment, ecg monitoring, portable medical device, remote patient monitoring, wearable technology

## Abstract

The electrocardiogram (ECG) is a non-invasive diagnostic tool that records the heart's electrical activity to detect and diagnose cardiac conditions. The 12-lead ECG remains the gold standard for initial evaluation due to its comprehensive waveform analysis. However, its bulkiness, need for precise electrode placement by trained personnel, and limitations for continuous or ambulatory monitoring render it impractical for routine use outside clinical environments. Portable and wearable ECG devices have emerged as viable alternatives, enabling accessible, real-time cardiac assessment in diverse settings. This study presents the design of a lightweight, wearable five-lead ECG device for non-invasive cardiac monitoring, featuring high-resolution signal acquisition and built-in protections against electrical interference to deliver clear, low-noise waveforms. In addition to a rechargeable battery for extended daily use and wireless Bluetooth transmission for seamless data sharing with clinicians, the device incorporates an onboard accelerometer that detects motion, posture, and sleep state to dynamically adjust filtering and further reduce motion noise. The work includes a schematic illustrating circuit integration and functionality, simulations demonstrating the filters’ effectiveness in attenuating unwanted signals from the electrodes, and a complete three-dimensional model of the printed circuit board with its enclosure. The proposed design demonstrates a feasible and efficient approach to compact, wearable ECG monitoring. By integrating an accelerometer for adaptive filtering, the system reduces motion noise and improves waveform reliability during daily activities. These findings support its potential use in continuous cardiac assessment outside clinical environments.

## Introduction

The electrocardiogram (ECG) is a cornerstone diagnostic technique in cardiology and an integral component of comprehensive medical evaluations. By capturing waveforms that reflect the heart's electrical activity, it enables the detection of various cardiac abnormalities, including myocardial ischemia, arrhythmias, and structural damage [1]. Currently, ECG monitoring extends across diverse settings, including hospitals, homes, and remote environments, supporting real-time assessment, patient self-management, and integration with physical activity tracking [2].

The 12-lead ECG remains the gold standard for cardiac assessment, providing a three-dimensional representation of the heart’s electrical activity. However, its bulk, requirement for trained personnel, and limited applicability outside clinical settings restrict its use for continuous or ambulatory monitoring [3].

There is a growing global demand for continuous health monitoring systems capable of measuring heart rate variability, enabling the early detection and treatment of cardiovascular diseases [4]. Continuous ECG recording anytime and anywhere can provide clinicians with evidence of cardiac abnormalities that may no longer be detectable in a 12-lead ECG obtained later during a medical appointment [5]. In recent years, to address these limitations, portable and wearable ECG technologies have emerged as a promising solution. These compact systems enable basic arrhythmia detection using a single lead [6], thereby simplifying operation and allowing use by individuals without specialized training.

These wireless ECG devices consist of a number of electrodes, an analog front-end (AFE), which amplifies and filters weak bioelectric signals, a data acquisition (DAQ), which converts the analog signals to digital form, a digital signal processing unit (DSP), which cleans and analyzes the signals, any form of wireless communication such as Bluetooth, Wireless, Infra-Red (IR), and a power management and energy supply unit [7].

Although numerous ECG devices are currently available, a major challenge remains: patient movement during daily activities introduces unwanted signal noise, which reduces monitoring accuracy and may lead to incorrect interpretations. Since these noises occur within the same frequency range as the ECG signal, they overlap with it, making it difficult to differentiate noise from the true cardiac waveform [8].

Recent advances in low-power electronics, wireless communication, and biomedical sensors have enabled the development of compact wearable systems capable of continuous cardiac monitoring outside clinical settings [8,9]. Building on these technological improvements, this design study presents the design and simulation of a lightweight, wearable five-lead ECG device that integrates adaptive filtering and motion detection. The system employs an onboard accelerometer to identify user movement and dynamically adjust filter parameters, thereby maintaining stable, low-noise ECG signals during everyday activities. This approach aims to enhance diagnostic reliability while providing a practical and accessible tool for remote cardiac assessment.

While prior wearable ECG devices, such as KardiaMobile (AliveCor, Inc., Mountain View, California, United States) and Zio Patch (iRhythm Technologies, Inc., San Francisco, California, United States), enable single-lead monitoring, they provide limited spatial resolution and lack motion-adaptive filtering, leading to signal corruption during activity. The design presented in this report addresses this by integrating a multi-lead ADS1293 front-end with adaptive accelerometer-based filtering for continuous, artifact-resistant monitoring.

## Technical report

Design approach

This device was developed to address the current limitations of portable, battery-powered ECG systems by providing high signal quality, compact design, and low cost. The goal is to create a solution that can be seamlessly integrated into daily life without causing significant discomfort or requiring extensive maintenance, while remaining practical for continuous, everyday use.

The design process commences with acquiring electrical signals from the electrodes, followed by processing through adjustable filters to eliminate extraneous noise. The refined signal is then directed to the primary integrated circuit for digitization, after which the data is transmitted via Bluetooth to a smartphone or compatible device, enabling subsequent sharing with healthcare providers for expert interpretation. To minimize motion-induced noise and ensure compliance with clinical standards for adult ECG interpretation (band-pass range 0.05-150 Hz) [10], an accelerometer-based adaptive filtering mechanism is implemented to dynamically adjust filter parameters according to detected movement.

**Figure 1:**
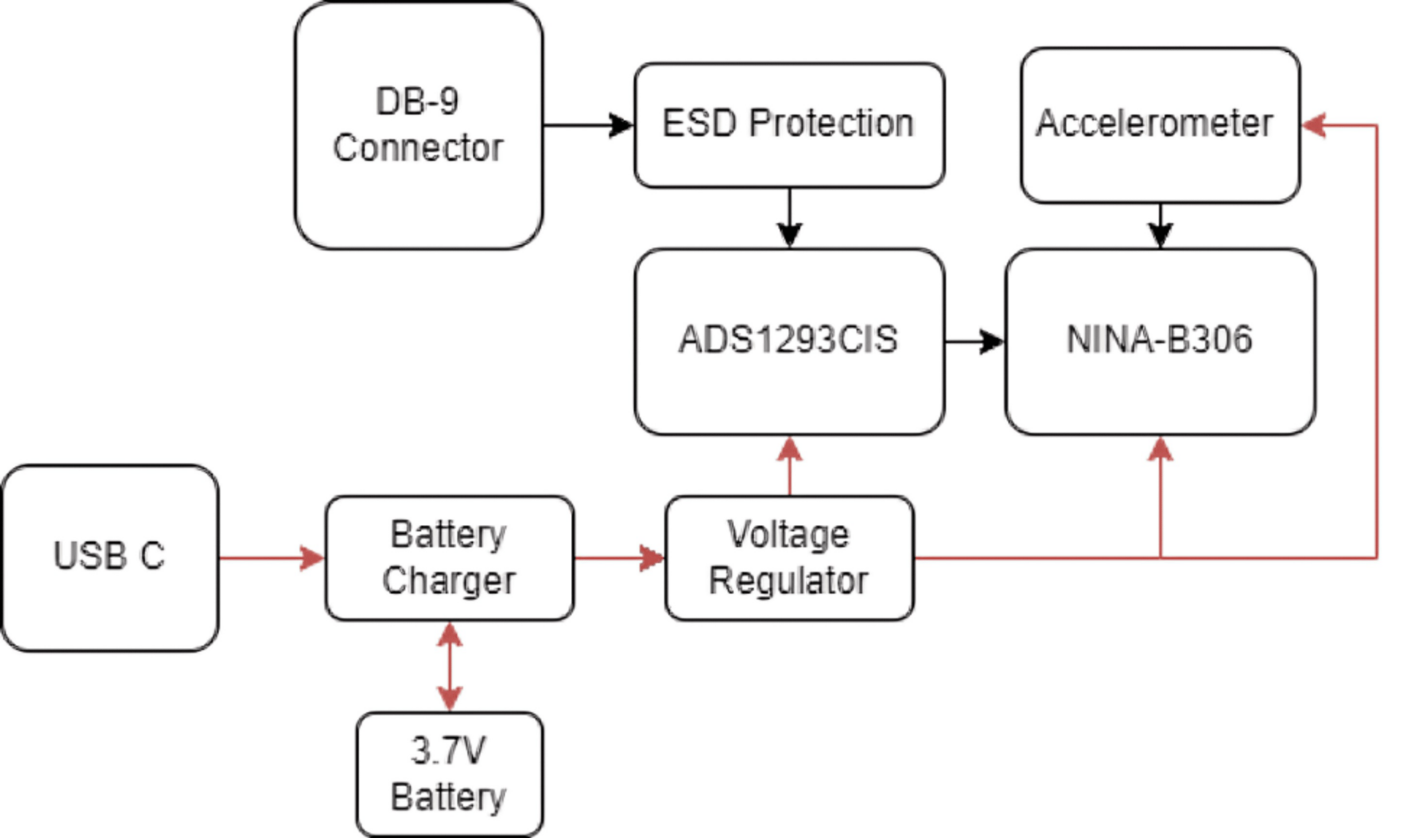
System architecture of the proposed portable ECG device This diagram illustrates the main functional blocks of the portable ECG monitoring device, including signal acquisition, processing, wireless Bluetooth transmission, and power regulation. Electrical signals from the electrodes pass through electrostatic discharge (ESD) protection and amplification stages before being digitized by the ADS1293 (Texas Instruments Incorporated, Dallas, Texas, United States) analog front-end. The NINA-B306 (u-blox Holding AG, Thalwil, Switzerland) controller manages Bluetooth Low Energy (BLE) communication, while the LIS2DW12TR (STMicroelectronics NV, Plan-les-Ouates, Geneva, Switzerland) accelerometer provides motion data for adaptive filtering. Image Credit: Author

The overall system architecture, illustrated in Figure 1, demonstrates the interaction between the signal acquisition, processing, communication, and power management modules.

Hardware components

The core component of the system is the NINA-B306 (u-blox Holding AG, Thalwil, Switzerland) Bluetooth controller, which facilitates wireless transmission of acquired ECG data to nearby compatible devices. This enables seamless forwarding to healthcare providers for expert analysis. Its low-power operation significantly extends battery life, allowing the device to function for multiple days on a single charge.

The ADS1293 (Texas Instruments Incorporated, Dallas, Texas, United States) serves as the primary analog front-end responsible for capturing the heart’s electrical signals. It amplifies and filters the weak biopotential signals detected by the electrodes, ensuring accurate and low-noise ECG measurements. Its compact design and minimal power consumption make it well-suited for integration into portable, battery-powered medical devices.

The LIS2DW12TR (STMicroelectronics NV, Plan-les-Ouates, Geneva, Switzerland) is a low-power, three-axis accelerometer integrated into the system to detect subtle body movements. The motion data it provides indicates how the filtering parameters of the ECG signal should be adjusted to reduce motion-related noise and maintain signal accuracy.

The device also includes a USB (Universal Serial Bus) Type-C port that serves a dual purpose for both charging and programming. It allows convenient recharging of the internal lithium battery while also providing a wired interface for firmware updates or device calibration. Integrating a universal connector simplifies maintenance and ensures compatibility with standard power adapters and computer interfaces.

To conceptually demonstrate the ADS1293’s filtering behavior, a simplified two-stage resistor-capacitor (RC) network was simulated in LTspice (Linear Technology Corporation, Milpitas, California, United States) to approximate its internal digital filter, focusing on a bandpass response.

Results

**Figure 2:**
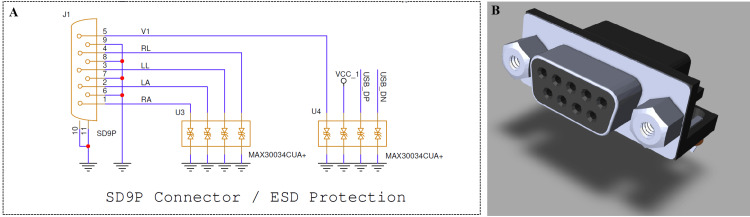
SD9P connector interface and ESD protection circuit (A) Schematic diagram showing the SD9P connector (J1) interface used for ECG electrode inputs: right arm (RA), left arm (LA), left leg (LL), right leg (RL), and precordial lead 1 (V1)  and the corresponding electrostatic discharge (ESD) protection circuits (U3 and U4). (B) Three-dimensional model of the SD9P female connector illustrating the mechanical design and pin layout used in the system. Image Credit: Author

As illustrated in Figure 2, the SD9P connector provides the primary interface between the ECG electrodes and the acquisition circuitry, ensuring electrical safety and stable biopotential signal transmission. All input lines are protected by MAX30034CUA+ (Analog Devices, Inc., Wilmington, Massachusetts, United States) ESD protection arrays to prevent damage from electrostatic discharges and voltage surges, and are intended to comply with relevant medical device safety standards.

**Figure 3:**
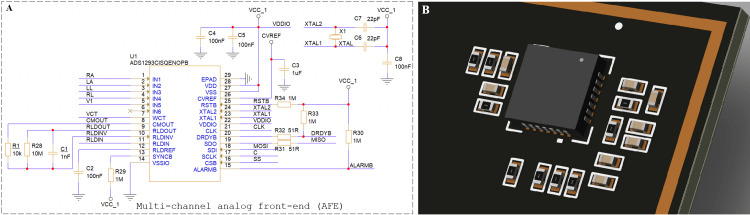
Multi-channel AFE and PCB implementation (A) Schematic of the ADS1293-based analog front-end (AFE) circuit used for multi-lead ECG acquisition. (B) Three-dimensional printed circuit board (PCB) rendering of the ADS1293 module layout. Image Credit: Author

The signals that come from the SD9P connector directly connect to the ADS1293 IC. The ADS1293 is a multi-channel AFE responsible for capturing and conditioning the electrical activity of the heart prior to digital processing. The circuit is centered around the ADS1293CISQENOPB, a specialized biomedical front-end designed for high-precision ECG acquisition. This integrated device combines multiple low-noise amplifiers, programmable filters, and high-resolution analog-to-digital converters (ADCs), allowing simultaneous monitoring of several ECG leads with minimal external components. Multi-channel AFE and PCB implementation is shown in Figure 3.

**Figure 4:**
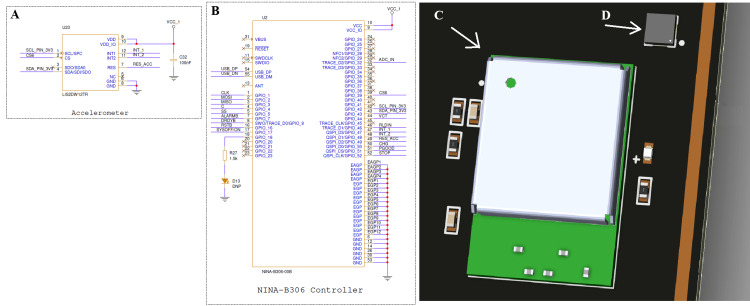
NINA-B306 Bluetooth controller and LIS2DW12 accelerometer integration (A) Circuit schematic of the LIS2DW12TR (STMicroelectronics NV, Plan-les-Ouates, Geneva, Switzerland) three-axis accelerometer used for motion detection and activity recognition. (B) Schematic of the NINA-B306 (u-blox Holding AG, Thalwil, Switzerland) Bluetooth Low Energy (BLE) controller, which serves as the primary processing and communication unit of the system. (C) Three-dimensional printed circuit board (PCB) rendering of the NINA-B306 module layout, showing compact component placement. (D) Close-up view of the LIS2DW12 accelerometer positioned near the NINA module to reduce signal path length and mechanical vibration noise. Image Credit: Author

Figure 4 illustrates the wireless communication and motion-sensing subsystem of the device, which combines the NINA-B306 Bluetooth controller with the LIS2DW12TR accelerometer for synchronized data acquisition and transmission. The NINA-B306 module enables both real-time processing and wireless connectivity in a compact and energy-efficient platform. Its multiple general-purpose I/O pins and serial interfaces allow seamless integration with the ADS1293 analog front-end and other peripheral sensors. The LIS2DW12TR, a three-axis low-power accelerometer, continuously measures motion and posture changes, providing valuable contextual information about patient activity. The motion data collected by the accelerometer is transmitted to the NINA-B306 controller, which in turn adjusts the internal filter configuration of the ADS1293 front-end to minimize motion artifacts and maintain ECG signal clarity during patient movement.

**Figure 5:**
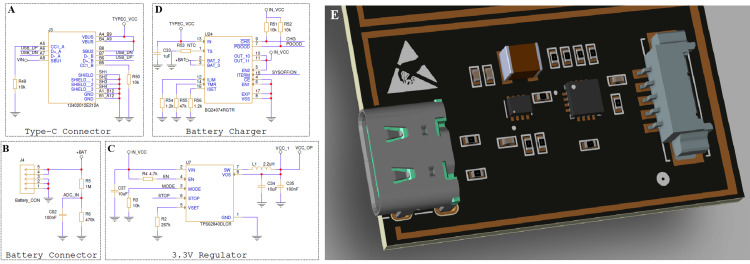
Power management subsystem including USB Type-C interface, battery charger, and voltage regulation stage (A) Circuit schematic of the Universal Serial Bus (USB) Type-C interface. (B) Battery connector circuit. (C) 3.3-volt switching regulator stage based on the TPS62840DLCR (Texas Instruments Incorporated, Dallas, Texas, United States). (D) Battery-charging circuit implemented using the BQ24074RGTR (Texas Instruments Incorporated) linear charger. (E) Three-dimensional printed circuit board (PCB) rendering of the power-management section, showing the placement of the USB-C port, charger integrated circuit (IC), and associated passive components. Image Credit: Author

Figure 5 illustrates the power management subsystem responsible for providing stable and efficient power to all components of the wearable monitoring device. The design integrates four key stages: a USB Type-C interface, a battery charger, a voltage regulator, and the battery connector (Figure 5A-5D). The Type-C connector allows both external power delivery and data communication, enabling device charging and firmware updates through a single port. The BQ24074RGTR (Texas Instruments Incorporated, Dallas, Texas, United States) integrated circuit functions as a linear battery charger with built-in power-path management, allowing the system to operate directly from USB power while simultaneously charging the lithium-ion cell. The TPS62840DLCR (Texas Instruments Incorporated) switching regulator efficiently converts the battery voltage to a stable 3.3 V supply required by the digital controller, analog front-end, and wireless communication modules. This regulator’s high conversion efficiency minimizes heat generation and extends battery life during continuous operation. The battery connector section includes a voltage-sensing divider that allows real-time monitoring of the battery’s voltage level through the controller’s ADC input. The adjacent 3D PCB rendering highlights the compact physical layout of the power stage, where short trace lengths and closely placed decoupling capacitors minimize power losses, electromagnetic interference, and noise coupling into the sensitive analog circuitry. Together, these design features ensure stable operation, safe charging, and prolonged battery autonomy, which are essential for continuous wearable health monitoring applications.

To quantify the expected power requirements of the proposed architecture, Table 1 summarizes the continuous-mode current draw and power consumption of the main subsystems, calculated from manufacturer datasheet values at 3.3 V operation.

**Table 1:** Power and performance budget of the proposed 5-lead ECG device Summary of the typical current draw and power consumption of the main hardware subsystems, calculated from datasheet specifications at 3.3 V operation.

Subsystem	Symbol	Typical current (mA)	Supply (V)	Power (mW)
ADS1293 AFE	I_ADS1293	0.273	3.3	0.901
NINA B306 BLE MCU	I_NINA	4	3.3	13.2
LIS2DW12 accelerometer	I_LIS2DW12	0.00038	3.3	0.001254
TPS62840 regulator	I_TPS62840	0.00006	3.3	0.000198
Total	I_total	4.27344	3.3	14.102352

Assuming a 1000 mAh single-cell lithium battery and continuous operation at the calculated 4.27344 mA load, the theoretical runtime is 234.0 hours (9.75 days).

**Figure 6:**
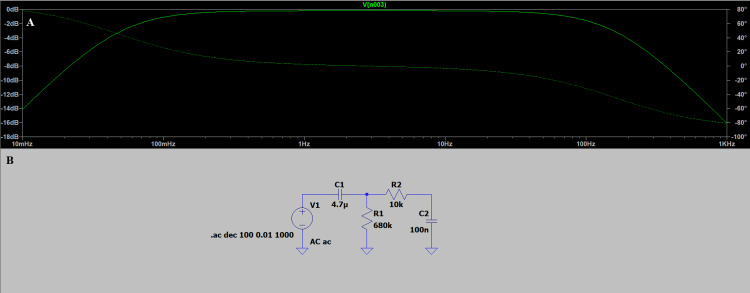
Simulated frequency response of a two-stage band-pass filter approximating the internal filtering behavior of the ADS1293 (A) Simplified analog model used to approximate the internal filtering architecture of the ADS1293 (Texas Instruments Incorporated, Dallas, Texas, United States) integrated circuit (IC). (B) Simulated frequency response obtained in LTspice (Linear Technology Corporation, Milpitas, California, United States). Image Credit: Author

To illustrate how the ADS1293 processes biopotential signals, a simplified two-stage RC network was simulated in LTspice as an analog approximation of its internal digital filtering architecture. The circuit includes a high-pass stage to remove baseline drift and a low-pass stage to attenuate high-frequency noise, producing a band-pass response. This conceptual model (Figure 6) demonstrates how the ADS1293 confines the ECG signal to the clinically relevant bandwidth, with approximate -3 dB cutoffs near 0.05 Hz and 150 Hz, along with attenuation exceeding -20 dB outside the passband. These results represent an educational approximation of the device’s filtering behavior.

**Figure 7:**
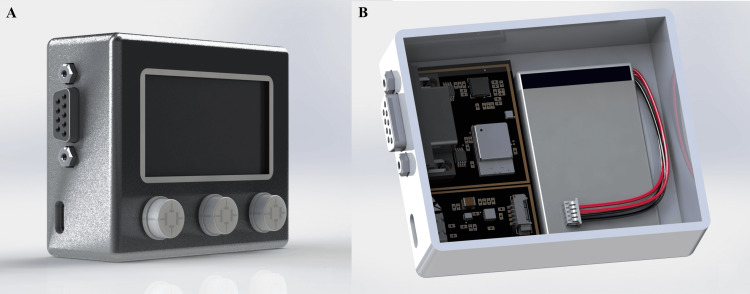
External and internal view of the portable ECG monitoring device (A) Outer enclosure showing display and interface connectors of the portable ECG monitoring device. (B) Internal assembly illustrating printed circuit board (PCB) and battery placement. Image Credit: Author

Figure 7 illustrates both the external and internal configurations of the developed portable ECG monitoring device. The compact enclosure (Figure 7A) houses a front-mounted display and three control interfaces for user interaction, alongside a DB-9 connector that serves as the main input for ECG electrode signals. A USB-C port positioned on the lower side allows both battery charging and firmware updates through a standard interface. The internal assembly (Figure 7B) reveals the organized placement of the main printed circuit boards containing the analog front-end, Bluetooth communication module, and power management circuitry. A rechargeable lithium battery is positioned adjacent to the electronics to maintain balance.

## Discussion

The proposed portable ECG monitoring device demonstrates the feasibility of integrating advanced electronic components into a compact, low-power system suitable for continuous cardiac assessment outside clinical environments. Incorporating the LIS2DW12TR accelerometer for adaptive filtering effectively mitigates motion artifacts, one of the primary challenges in wearable ECG systems. By combining the ADS1293 analog front-end with the NINA-B306 Bluetooth controller, the design achieves high-fidelity signal acquisition and reliable wireless transmission while maintaining low energy consumption.

Compared to existing single-lead wearable ECG devices such as the KardiaMobile [3], which provide basic arrhythmia screening with moderate diagnostic accuracy for atrial fibrillation (AF) but limited capability for complex rhythm analysis due to reduced spatial resolution, the proposed five-lead configuration offers enhanced diagnostic potential. Multi-lead wearables, including six-lead patches, have demonstrated superior sensitivity (up to 99%) and specificity (up to 97%) for AF detection compared to single-lead smartwatches (69-86% sensitivity). The presented five-lead approach bridges the gap between the simplicity of single-lead systems and the comprehensive diagnostic coverage of 12-lead ECGs, enabling identification of conditions such as AF, atrioventricular blocks, and sick sinus syndrome, where accurate waveform morphology is essential for early detection and prevention of complications like stroke or sudden cardiac death [11].

Although this work primarily focuses on electronic design and simulation, further validation using real-world ECG signals is required to confirm clinical performance. Future efforts will include experimental testing under varying activity levels to quantify signal-to-noise improvements and refine adaptive filter parameters. Additionally, extended battery life and patient comfort will be evaluated to optimize long-term usability. Overall, this study establishes a robust foundation for the continued development of affordable, compact, and intelligent ECG monitoring systems aimed at improving remote cardiac diagnostics and patient outcomes.

## Conclusions

This report presents the design and simulation of a compact, low-power five-lead ECG monitoring device optimized for continuous cardiac assessment outside clinical environments. By integrating adaptive filtering through an onboard accelerometer, the system effectively reduces motion artifacts and enhances waveform stability.

Although hardware fabrication and in-vivo testing remain as future steps, the presented design establishes a solid foundation for developing affordable, reliable, and intelligent wearable ECG systems suitable for remote healthcare applications. Future work includes prototype fabrication and validation using ECG simulators and clinical testing data.
